# Early diagnosis program for multimodal primary open-angle glaucoma based on clinical process

**DOI:** 10.3389/fopht.2026.1975341

**Published:** 2026-10-21

**Authors:** Jingyi Ma, Bin Lv, Yuanyuan Li, Pan Fan, Xu Zhao, Yingtao Zhang, Huiping Yuan

**Affiliations:** 1 The Department of Ophthalmology, The Second Affiliated Hospital of Harbin Medical University, Harbin, Heilongjiang, China; 2 School of Computer Science and Technology, Harbin Institute of Technology, Harbin, Heilongjiang, China

**Keywords:** deep learning, early diagnosis, machine learning, multimodal, primary open-angle glaucoma

## Abstract

**Background:**

Glaucoma is among the leading causes of blindness. It is important to detect glaucoma at early stages. Using structural and functional examinations to identify primary open-angle glaucoma (POAG) is critical. Computer-aided diagnosis of glaucoma requires multimodal data to identify an accurate and explainable model for early glaucoma diagnosis.

**Methods:**

In this study, 301 multimodal data points were collected from the ophthalmology department at the Second Affiliated Hospital of Harbin Medical University. Data regarding fundus photography (FP), retinal nerve fiber layer thickness (RNFLt), intraocular pressure (IOP) values, visual field examination parameters and age were obtained. A powerful deep learning network was used to segment the optic nerve head obtained from the FPs. Machine learning classifiers (MLCs) were adopted to obtain the final classification results using 3 datasets: early glaucoma eyes, suspected glaucoma eyes, and healthy eyes; moreover, the diagnostic results of glaucoma specialists and general nonglaucoma ophthalmologists were collected.

**Results:**

The overall area under the curve was 0.9858. The accuracy values of the deep learning- machine learning system, the glaucoma specialist group, the general ophthalmologist group were 0.9478, 0.9493, 0.8725, respectively.

**Conclusions:**

The algorithm delivers superior results in the early diagnosis of POAG. This study’s hybrid deep learning-machine learning framework can effectively assist with clinical decision-making for early POAG.

## 1 Introduction

Glaucoma is among the leading causes of blindness (1, 2). Primary open-angle glaucoma (POAG) is characterized by chronic progressive optic nerve damage, which manifests as thinning of the retinal nerve fiber layer (RNFL) and progressive visual field damage (3, 4). Due to the risk of irreversible blindness, the early diagnosis of POAG is crucial for maintaining the visual function of glaucoma patients (5). In developing countries, approximately 50%–90% of patients with POAG are not aware of symptoms from the early stage (6). However, an effective method is still needed to diagnose early POAG at the population level (7). With the continuous development of computer technology and image segmentation technology, research on the use of machine learning and deep learning to diagnose ocular diseases is growing (8, 9). Previous studies have attempted to classify manual features through machine learning classifiers (MLCs) (10–13). Researchers have used deep learning (DL) methods to identify glaucoma or suspicious glaucoma through fundus photography (FP) and optical coherence tomography (OCT). However, when structural changes occur, the disease has already progressed to a severe stage (14).

According to the European Glaucoma Society Terminology and Guidelines for Glaucoma (15), the early clinical diagnosis of POAG is mainly based on characteristic changes in the optic disc and visual field changes, which requires structural and functional examinations. Structural examinations primarily rely on FP and OCT (16), which is defined as a noninvasive high-resolution biological imaging tomography of the retina that provides quantitative analysis. Through the use of OCT scanning, retinal nerve fiber layer defects, retinal ganglion cell complex thinning and optic disc changes can be detected (17, 18). Functional assessment mainly relies on standard automated perimetry (SAP) (19), which also determines the progression of the disease (20). Therefore, to improve the diagnostic accuracy, the combination of structural and functional tests is needed (21, 22).

The early diagnosis of glaucoma requires comprehensive consideration of auxiliary examination results from various aspects. Therefore, the screening of a quantity of high-quality data and development of a more systematic and explicable diagnostic model are challenging.

When considering the clinical process of glaucoma diagnosis, we attempted to develop a hybrid deep learning-machine learning framework to diagnose POAG from an early stage and compared the obtained data with the diagnostic accuracies of glaucoma specialists and general ophthalmologists.

## 2 Methods

### 2.1 Study population and data source

We conducted a series of studies using retrospective cases at the Second Affiliated Hospital of Harbin Medical University. Cases were collected from the ophthalmology department with cases involving early POAG eyes, suspected POAG eyes, and healthy eyes. This research followed the tenets of the Declaration of Helsinki.

All of the subjects met the following two inclusion criteria: best corrected visual acuity ≥0.5 logMAR, refractive error between +3 and -6 diopters, open angles on gonioscopy and reliable SAP exams, along with false-positive errors, false-negative errors and fixation losses less than 15%. Patients with other retinal diseases, secondary glaucoma, other causes of optic neuropathy, a history of intraocular surgery, or systemic diseases such as diabetes and hypertension were excluded.

The inclusion criteria for the early POAG group (23) involved a glaucomatous optic disc lesions (defined as a cup-disk ratio >0.6 or a cup-disc ratio asymmetry >0.2, focal defects of the neuroretinal rim, or disc hemorrhage), glaucomatous visual field damage (24) and an MD value of -6 dB or better. An intraocular pressure (IOP) fluctuation (maximum IOP value minus minimum IOP value) ≥8 mmHg 24 hours before the use of IOP-lowering drugs was considered supportive evidence for POAG inclusion (25).

The inclusion criteria for the POAG suspect group (26) involved an increase in the suspicious cup-to-disk ratio and/or a measurement of intraocular pressure >21 mmHg recorded at least twice (after corneal thickness correction), OCT fiber layer scanning suggesting RNFL thinning, and no observed visual field lesions characteristic of glaucoma. The exclusion criteria were the same as those utilized for the earlier POAG group.

The inclusion criteria for the healthy group involved an intraocular pressure between 10 and 21 mmHg (after corneal thickness correction), a lack of family history of glaucoma, a normal visual field, or a normal optic disc.

All of the participants underwent a comprehensive ophthalmologic examination, including visual acuity measurements, optometry tests, slit lamp microscope examinations, gonioscopy tests, central corneal thickness measurements, IOP tests, SAP tests (SITA FAST 24-2, size III stimulus, Allergan Humphery-750, Carl Zeiss Inc., Dublin, CA), and OCT tests (Spectralis, Heidelberg, Germany). The intraocular pressure of each patient was measured three times by using a noncontact tonometer, and the average value was obtained. The IOP value of the POAG patients was measured under IOP-lowering medication.

A fundus photograph was obtained without mydriasis (Canon CR4–45 NM, Japan). All of the participants underwent SAP tests at least twice, with the first test being utilized as a trial test. Reliable visual field examination results were selected. SAP parameters were obtained and included the visual field index (VFI), mean deviation (MD), and pattern standard deviation (PSD). All OCT images were obtained by ophthalmologists with more than 5 years of experience in OCT scanning. No mydriasis was needed. Scanning was performed with a diameter of 3.46 mm around the optic disc. The RNFL thickness (RNFLt) values of 6 quadrants (including temporal, nasal, superior temporal, superior nasal, inferior temporal, and inferior nasal quadrants) were obtained. The time interval between all examinations for the same patient did not exceed 1 week.

### 2.2 The DL-ML framework

Three glaucoma specialists (with more than 5 years of experience) manually marked the OD and OC in each photograph (with patient information being hidden). A senior specialist with more than 10 years of experience in glaucoma evaluation subsequently reviewed the data. A deep learning method was used to segment the optic nerve head based on the ophthalmologists’ annotations (**Figure 1**). The DL computed the vertical cup-to-disc ratio (VCDR), the horizontal cup-to-disc ratio (HCDR), and the width of the four rims (including the inferior, superior, temporal, and nasal rims). MLCs were used to obtain the final classification results. Based on the abovementioned examinations, 13 features were used to test and verify the MLCs, including age, 6-sector RNFLt, VFI, MD, PSD, mean IOP value, HCDR, and VCDR obtained via FP segmentation (**Figure 2**).

**Figure 1:**
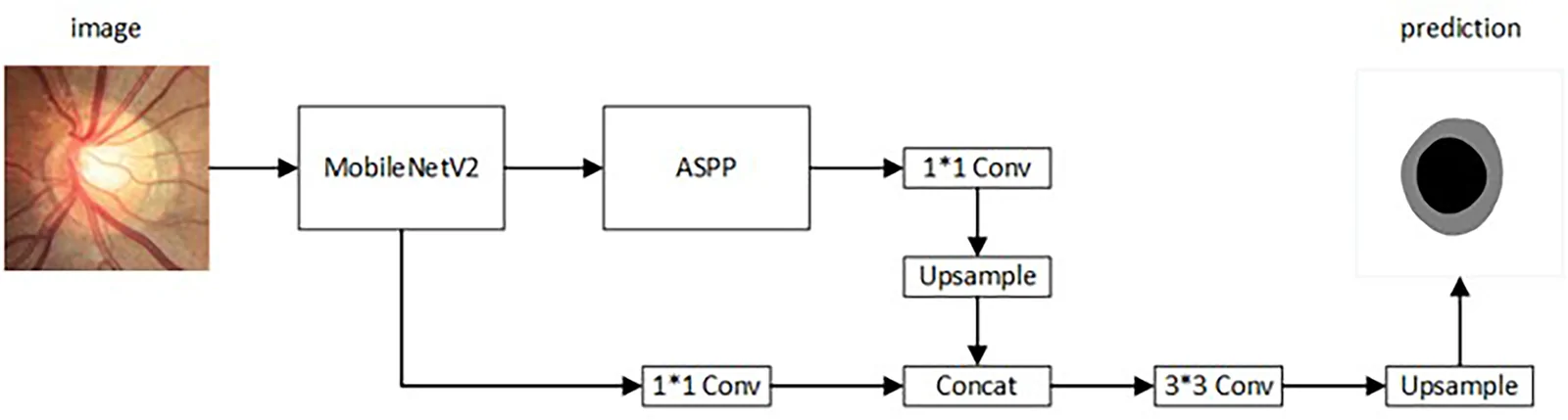
Deep learning system for the segmentation of fundus photographs. ASPP, atrous spatial pyramid pooling; Conv, convolution.

**Figure 2:**
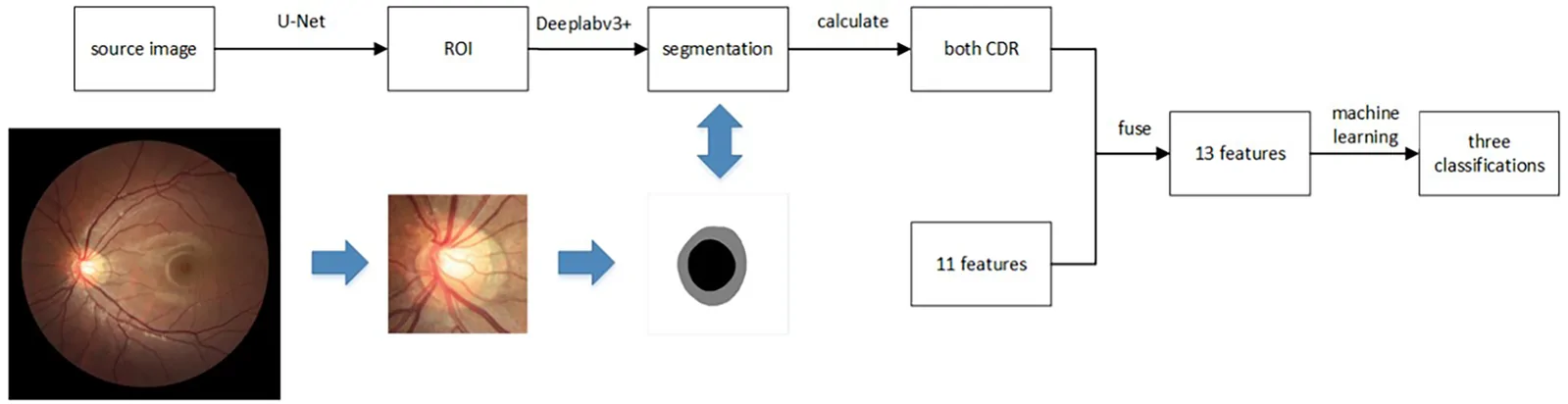
An overall framework of the diagnostic system. ROI, region of interest; CDR, cup-to-disc ratio.

The entire software environment was represented by Python version 3.6 (Anaconda, Inc.). The segmentation framework was implemented in Python based on Keras (27), with the TensorFlow backend being utilized. We used the Adam (28) optimizer and set the initial learning rate to 1e-3. Two NVIDIA GeForce RTX 2080 GPUs were used to support the computation with a mini-batch size of 4. In total, we trained the segmentation network with 120 epochs in each folder. The classifiers were mainly programmed in the Sklearn packages. The main segmentation code was based on Wang’s research (29). In our study, we used the segmentation network of deeplabv3plus (30) with a backstone network of MobileNetV2 (31). The region of interest (ROI) was defined as the region that included the optic disc. We located and cropped the images to a size of 512*512. ROI images were fed into the segmentation network; additionally, we trained the DLC and obtained the mask of the ROI images. FP sets were tested with 8-fold cross validation (training: validation: test=6:1:1). Finally, we marked the edge of the mask on the original images to show the performance. Afterward, we trained and tested the following algorithms: support vector machine (SVM) (32), random forest (RF), logistic regression (LR) and artificial neural network (ANN). Moreover, the ANN had one hidden layer that included 512 units. The data preprocessing process standardizes the 13-dimensional feature data. Healthy eyes, suspected glaucoma eyes, and early glaucoma eyes were assigned class labels of 0, −1, and 1, respectively. Specifically, the mean and standard deviation of each feature are calculated from the training data, and each feature is then standardized using these statistics so that it has a mean of approximately 0 and a standard deviation of approximately 1. This puts all features on a similar scale and reduces the effects of differences in their units and numerical ranges. All 4 machine learning methods were tested with 5-fold cross validation. In each iteration, four folds were used to train the model, while the remaining fold was used for validation. The validation data were not involved in model fitting, so the model had no access to their features or labels during training. A random seed was specified to support reproducibility. The input features were predefined based on their clinical relevance to glaucoma assessment. Instrument-derived measurements routinely considered during clinical diagnosis were combined with cup-to-disc ratios obtained from optic disc and cup segmentation. Fundus photographs were used for segmentation, but high-dimensional image features learned directly from the photographs were not included in the final classifier, to facilitate clinical interpretation. The accuracy and other evaluation metrics were then calculated on the validation fold. This process was repeated five times, with a different fold used for validation each time, and the final accuracy was reported as the average of the five validation accuracies. Each fold preserved approximately the class distribution of the full dataset. Cross-validation was performed at the record level without enforced patient-level grouping.

The DL-ML framework was evaluated by using the mean intersection over union (Miou), sensitivity, specificity, accuracy, F1 score, Mathews correlation coefficient (MCC), and area under the receiver operating characteristic curve (AUROC) parameters (33). More specifically, the AUROC was calculated by averaging the performance of the 5-fold cross validation. The number of correct categories was summed and divided by the total number of samples in each of the three categories to determine the accuracy value. To calculate the sensitivity, specificity, F1 score, and MCC value, the data were classified as follows: group 0 established POAG suspects as positive samples, as well as early POAG and healthy eyes as negative samples; group 1 established healthy eyes as positive samples, as well as early POAG and POAG suspects as negative samples; and group 2 established early POAG as positive samples, as well as POAG suspects and healthy eyes as negative samples. Three matrices were generated, and the results were calculated by averaging the performance of these three groups. The feature importance was determined according to RF.

### 2.3 Clinician diagnosis

Three matrices were generated, and the results were calculated by averaging the performance of these three groups. Three glaucoma specialists (group A) and three general ophthalmologists (group B) with at least 5 years of clinical experience were chosen to diagnose all of the patients. Complete examination results with hidden clinical information were obtained for each group. Each clinician reviewed OCT reports, visual field reports, and fundus photographs with diagnostic labels removed, together with 11 features including age, IOP, six-sector RNFL thickness, VFI, MD, and PSD. Cases were diagnosed into three groups of results: 1 (healthy eye), 2 (POAG suspects), and 3 (early POAG). The average accuracy of each group was calculated according to the diagnostic test and compared to that of the DL-ML framework.

### 2.4 Statistical analysis

Differences among the three groups were compared via Welch ANOVA performed by using SPSS (version 22.0). Characteristics were reported as mean ± standard deviation, and P < 0.05 was considered statistically significant. Interobserver agreement among the three clinicians within each group was assessed using unweighted Fleiss’ kappa for the three diagnostic categories.

## 3 Results

A total of 301 eye-visit records of 176 patients were included in the study. The demographic characteristics of the patients are summarized in **Table 1**, and the statistical characteristics of the subjects’ data are shown in **Table 2**. Each group demonstrated 13 features, and each feature (except age) was observed to be significantly different among the three groups (P < 0.05). The values of the VFI, MD, and PSD did not significantly differ between the healthy group and the suspected group. Further pairwise comparisons of these certain features are shown in **Figure 3**.

**Table 1:** Demographic characteristics of the patients.

Characteristics	Early POAG	POAG suspects	Healthy eyes
Subjects	45	69	62
Eye-visit records	99	85	117
Gender (% female)	43.43	70.94	64.10

**Table 2:** Statistical characteristics of the subject data.

Characteristics	Early POAG (n=99)	POAG suspects (n=85)	Healthy eyes (n=117)	P value*
Age, Mean (SD), y	40.16 ± 14.87	39.19 ± 16.04	39.39 ± 14.07	0.892
IOP, Mean (SD), mmHg	18.18 ± 4.48	18.39 ± 3.59	16.98 ± 3.01	0.012
MD, Mean (SD), dB	-3.92 ± 1.32	-2.72 ± 2.05	-2.59 ± 1.70	<0.001
PSD, Mean (SD), dB	3.52 ± 1.75	2.08 ± 1.25	1.76 ± 0.67	<0.001
VFI, Mean (SD), %	0.94 ± 0.37	0.97 ± 0.31	0.98 ± 0.21	<0.001
cpRNFLt, Mean (SD), μm				
Temporal	62.58 ± 17.93	77.47 ± 18.33	83.92 ± 17.20	<0.001
Nasal	49.25 ± 18.57	58.71 ± 17.22	70.96 ± 15.23	<0.001
Temporal Superior	89.17 ± 29.25	115.11 ± 25.13	144.97 ± 19.30	<0.001
Nasal Superior	70.34 ± 30.72	93.87 ± 26.36	119.63 ± 23.36	<0.001
Temporal Inferior	95.11 ± 29.96	127.22 ± 22.95	155.58 ± 19.44	<0.001
Nasal Inferior	78.20 ± 23.10	96.06 ± 25.88	118.49 ± 24.73	<0.001
CDR computed by DLCs, Mean (SD)				
HCDR	0.75 ± 0.07	0.67 ± 0.07	0.56 ± 0.09	<0.001
VCDR	0.75 ± 0.06	0.66 ± 0.08	0.52 ± 0.09	<0.001

IOP, intraocular pressure; MD, mean deviation; PSD, pattern standard deviation; VFI, visual field index; cpRNFL, circumpapillary retinal nerve fiber layer; HCDR, horizontal cup-to-disc ratio; VCDR, vertical cup-to-disc ratio. *Welch ANOVA, p<0.05 indicates a statistically significant difference.

**Figure 3:**
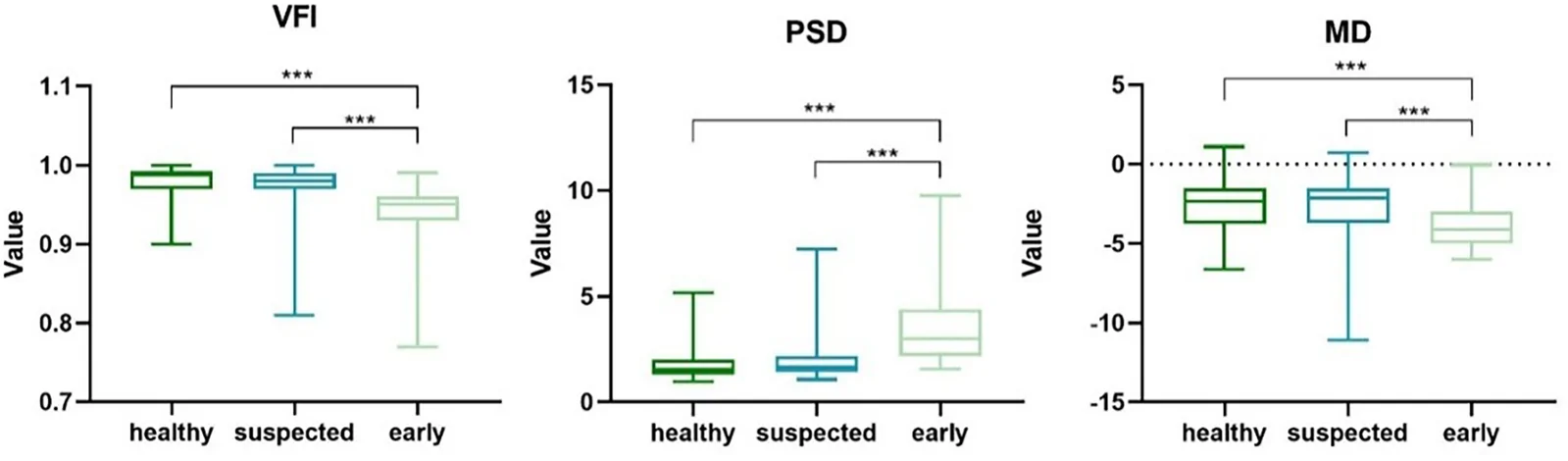
Multiple comparisons of visual field among healthy, suspected POAG, and early POAG eyes. Abbreviations: MD, mean deviation; PSD, pattern standard deviation; VFI, visual field index. ***Significant at the 1% level.

The mean values of optic nerve head recognition were 0.9342 and 0.7950, respectively. The ROC curves of the four MLCs are shown in **Figure 4**, and the other evaluation parameters are shown in **Table 3**. The SVM achieved the best AUC value (0.9858), as well as the best accuracy, F1 score and MCC results. The diagnostic accuracy of the glaucoma specialists was 0.9493, and that of the general ophthalmologists was 0.8725. The diagnostic accuracy value of the SVM with the highest AUC value was similar to that of the glaucoma specialists. The accuracies of the LR, RF, and ANN were 0.9130, 0.9217, and 0.9130, respectively. The average accuracies of glaucoma specialists and the general ophthalmologists were 0.9493 and 0.8725. Fleiss’ kappa was 0.7708 (95% CI, 0.7220–0.8169) among glaucoma specialists and 0.5633 (95% CI, 0.5054–0.6186) among general ophthalmologists. The accuracy results for the ophthalmologists are summarized in **Table 3**.

**Figure 4:**
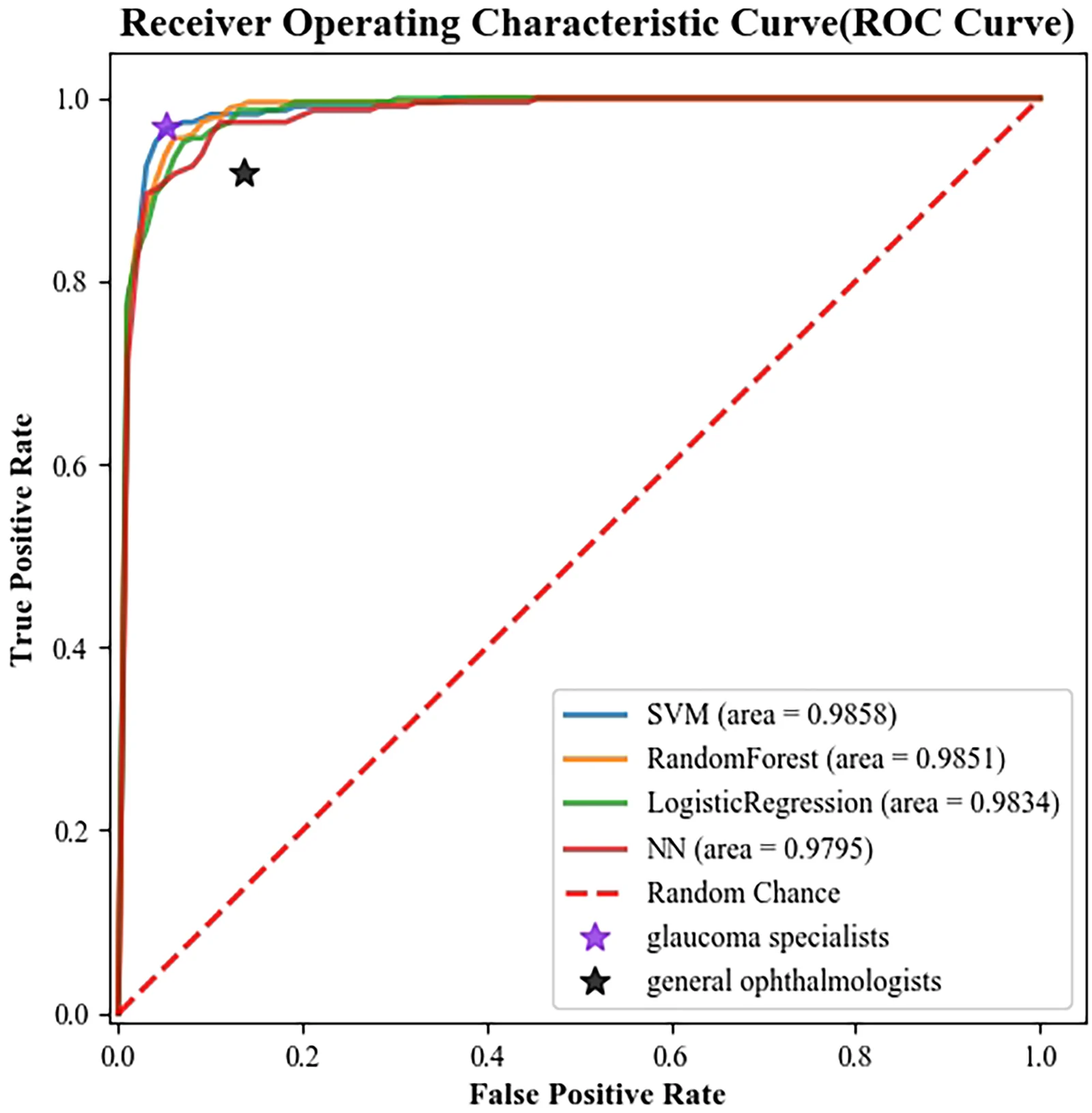
ROC curves for different MLCs.

**Table 3:** Performance of different machine learning classifiers and clinicians.

Approach	Accuracy (95% CI)	Sensitivity (95% CI)	Specificity (95% CI)	F1 score	Sensitivity at 80% specificity	Sensitivity at 90% specificity	AUC (95% CI)	MCC
SVM	0.9478 (0.9114-0.9644)	0.9577 (0.9236-0.9771)	0.9710 (0.9584-0.9844)	0.9513	0.9714	0.9570	0.9858 (0.9724-0.9961)	0.9255
RF	0.9217 (0.8903-0.9469)	0.9551 (0.9207-0.9791)	0.9476 (0.9329-0.9607)	0.9268	1.0	0.9570	0.9851 (0.9788-0.9950)	0.8903
LR	0.9130 (0.8613-0.9293)	0.9365 (0.8990-0.9583)	0.9521 (0.9327-0.9670)	0.9197	0.9857	0.9714	0.9834 (0.9680-0.9914)	0.8807
ANN	0.9130 (0.8757-0.9339)	0.9281 (0.8996-0.9500)	0.9588 (0.9403-0.9690)	0.9206	0.9143	0.9143	0.9795 (0.9626-0.9865)	0.8823
general ophthalm-ologists	0.8725	0.9194	0.8632	NA	NA	NA	NA	NA
glaucoma specialists	0.9493	0.9693	0.9475	NA	NA	NA	NA	NA

CI, confidence interval; SVM, support vector machine; RF, random forest; LR, logistic regression; ANN, artificial neural network; AUC, area under the receiver operating characteristic curve; MCC, Matthew’s correlation coefficient; and NA, not applicable.

The feature importance is shown in **Figure 5**. The top five features, including the VCDR (0.15), RNFLt-TI (0.12), RNFLt-TS (0.12), HCDR(0.10), and PSD (0.09), contributed the most to the algorithm. Meanwhile, we compared the SVM using all available predictors with models using individual groups and models omitting one group at a time. The mean AUC was 0.9858 for the full model, compared with 0.9756 for OCT alone, 0.8569 for CDR measurements, 0.7761 for visual-field parameters, and 0.7817 for age and mean IOP combined (**Supplementary Table S1**). Removing OCT parameters reduced the AUC to 0.9510. Consistent with feature importance showed by RF, these findings suggest that the changes in the ocular structure make a greater contribution to the models.

**Figure 5:**
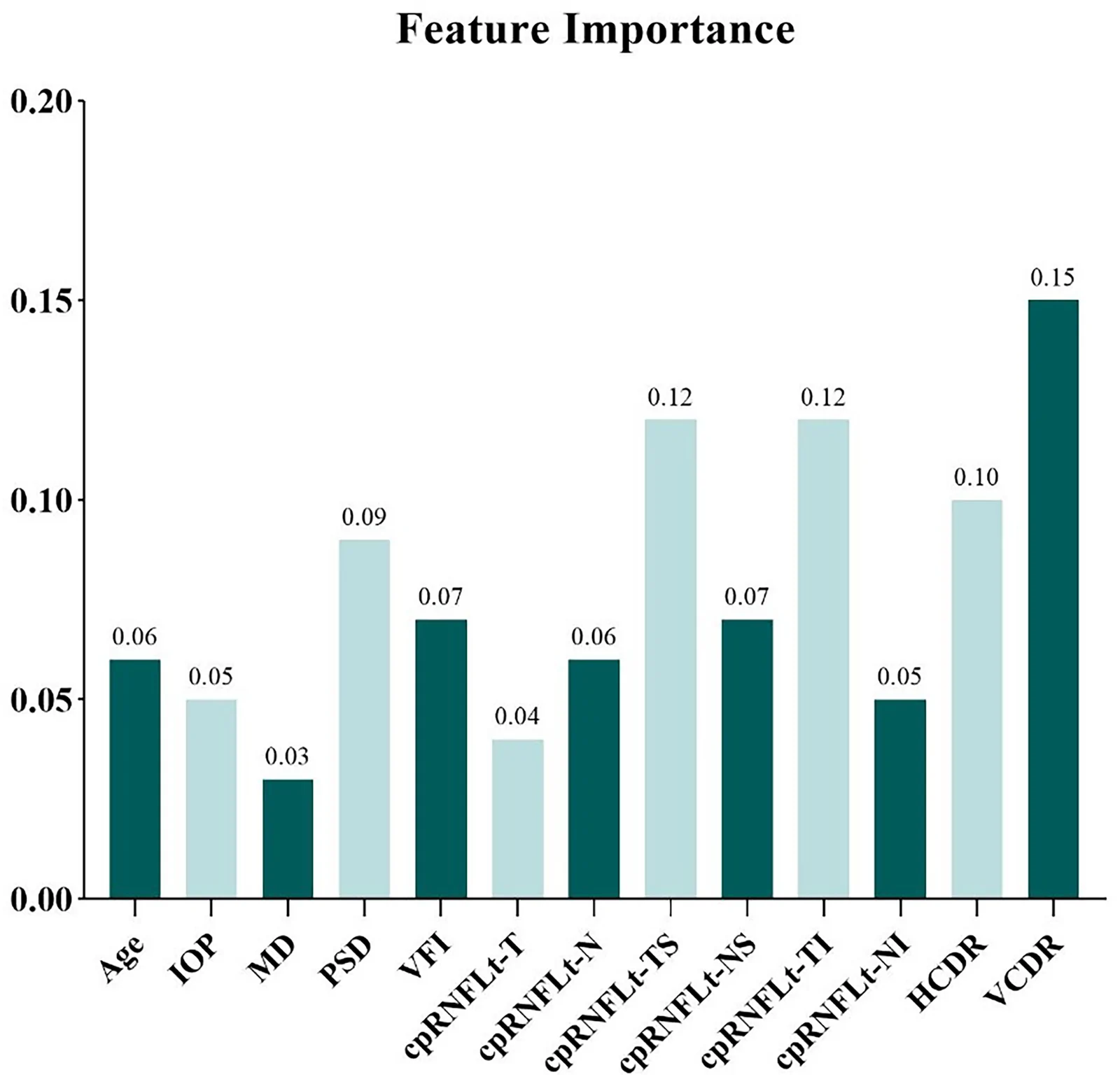
Feature importance shown by random forest. Abbreviations: IOP, intraocular pressure; MD, mean deviation; PSD, pattern standard deviation; VFI, visual field index; cpRNFL, circumpapillary retinal nerve fiber layer; HCDR, horizontal cup-to-disc ratio; VCDR, vertical cup-to-disc ratio.

## 4 Discussion

Glaucoma has become the leading cause of blindness. The early diagnosis of POAG is crucial for maintaining visual function. In a previous study of rural areas of East China (34), more than 90% of POAG cases were undetected in the early stage, and nearly 30% of subjects with glaucoma were blind in at least one eye. Therefore, we attempted to obtain high-quality multimodal data to develop an algorithm to aid primary hospitals in thoroughly diagnosing POAG from an early stage. In our study, we introduced a diagnostic model based on clinical processes to distinguish early POAG, POAG suspects and healthy eyes; moreover, we compared the diagnostic results of clinicians.

Previous attempts have been made to combine structure and function to detect POAG. Studies performed by Raza et al. (35) and Leonardo et al. (36) combined OCT and SAP results; the obtained results were better than those obtained by analyzing OCT results alone, thus indicating that structural and functional combined models yield better diagnostic results. Kim et al. (37) applied the random forest algorithm to detect glaucoma. The patient’s age, IOP, central corneal thickness (CCT), average RNFLT, glaucoma hemifield test (GHT), MD, and PSD data were recorded; moreover, the MLCs were trained, and the AUC was observed to be 0.979. However, the inclusion of advanced POAG patients in this study may significantly improve the accuracy of the results. A thorough examination of the optic disc is necessary in clinical practice. Therefore, in our study, we chose to simultaneously use structure, function, and intraocular pressure to diagnose early POAG and compared both POAG suspects and healthy eyes. Moreover, we used multimodal features to train and test a DL-ML framework and obtained satisfactory results. Another reason for obtaining a high AUC is that several model inputs, including RNFL thickness, visual field parameters, IOP, and CDR, overlap with the clinical information used to define the diagnostic groups. This overlap introduces a risk of incorporation bias and may contribute to the high observed AUC. Therefore, our model should be more utilized as an aid for clinical decision-making.

Our study has other strengths. First, due to black box effects, previous studies are difficult to explain to clinicians. In our study, feature importance can be provided by the RF and SVM, which makes the model more understandable to ophthalmologists. The feature importance demonstrated by the RF is highly consistent with clinical diagnosis. Furthermore, patients with a greater VCDR exhibit a greater risk of early-stage POAG (38). According to previous studies (39, 40), defects in the superior and inferior parts of the RNFL often occur in early POAG because of the anatomy of the retinal nerve fibers. Studies have proven that the importance of RNFL local thickness features is the same as that of POAG clinical manifestations (41). Moreover, previous studies have demonstrated that during the occurrence and development of early POAG, the change in the MD value of the visual field is relatively stable, whereas the RNFL exhibits relatively rapid thinning (42). PSD is more valuable for the diagnosis of early POAG as an indicator for the evaluation of local visual field defects (43). Therefore, RNFL thickness-related features and PSD values are greater than those of MD in the early clinical diagnosis of POAG. According to Kim’s study (37), the average RNFL thickness, intraocular pressure, PSD and MD strongly contributed to their model, which is consistent with the results of our study.

Second, studies have demonstrated that high myopia is among the high-risk factors for POAG (44). In our study, we excluded patients with high myopia and avoided RNFL thinning due to refractive factors, thus obtaining more reliable results.

The diagnostic accuracy of general ophthalmologists (0.8725) was lower than that of the MLCs. Interestingly, studies comparing the diagnostic ability between AI and ophthalmologists often demonstrate better performance for AI, which is similar to our results (36, 45). However, this system is intended primarily to support glaucoma screening and clinical decision-making in ophthalmic settings where multi-modal examinations are available. By integrating these examinations, the system may help identify individuals who require further glaucoma evaluation and assist ophthalmologists in distinguishing early POAG, POAG suspects, and healthy eyes.

Several examples of good and poor segmentation performance are shown in **Figure 6**. These findings suggest that small cups or notches may result in poor segmentation results. However, the results of the DLC segmentation are credible and provide relatively accurate measurements of the CDR value.

**Figure 6:**
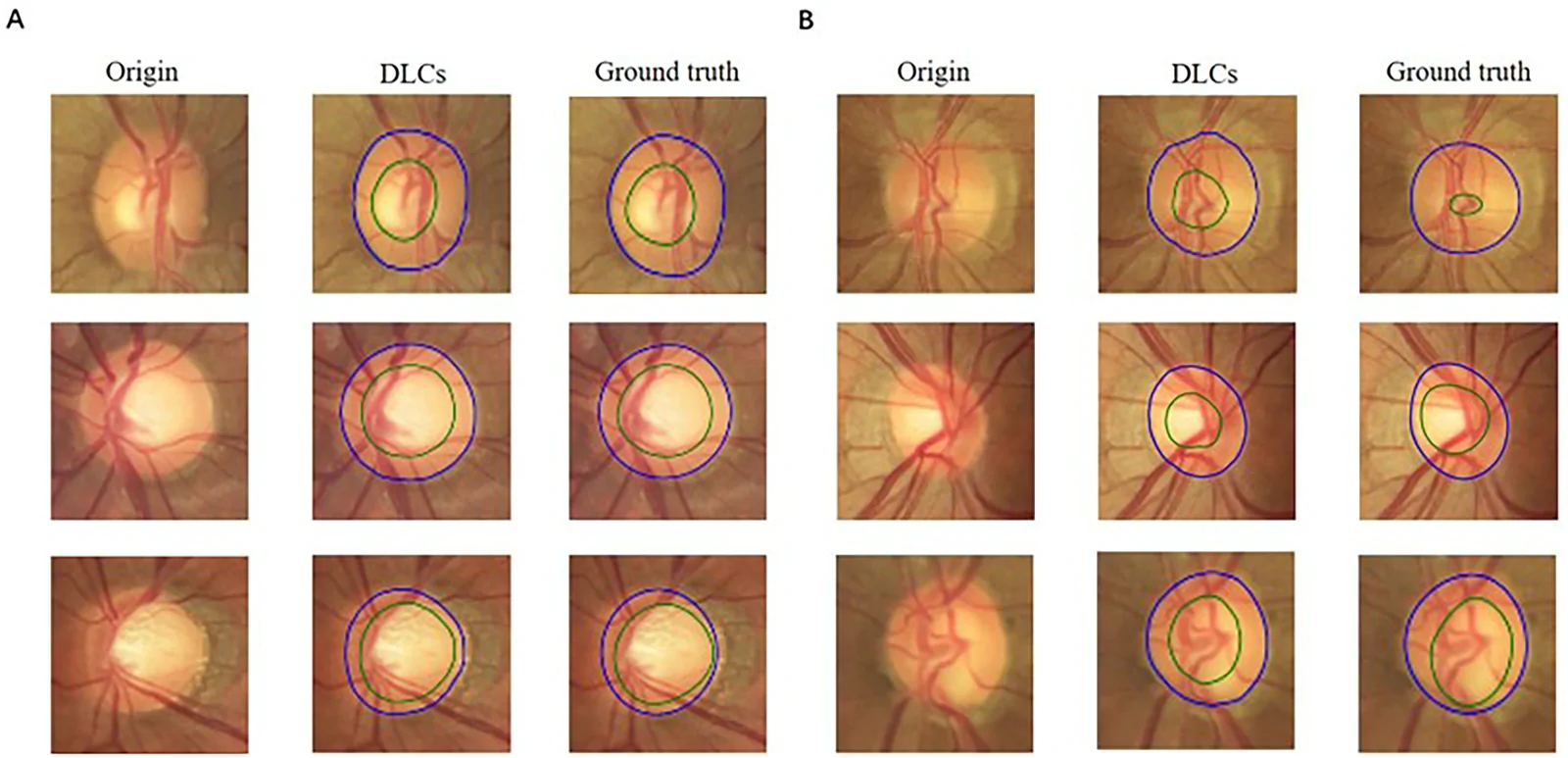
Examples of segmentation. **(A)** good performance and **(B)** poor performance. Each row presents one example. The green and blue contours indicate the boundaries of OC and OD, respectively. DLCs, deep learning classifiers.

One of the limitations of this study is that we included only Chinese patients from single center, and the number of patients in the training set was relatively small. Further studies with larger sample sizes are needed. The differences in imaging equipment, patient populations, disease severity, and clinical procedures among different hospitals result in variations in the collection of results. Therefore, the results obtained in this cohort should not be directly considered applicable to other medical institutions. It is necessary to conduct multi-center external validation to determine whether the model needs to be recalibrated or updated before clinical application. Another limitation of our study is the potential for incorporation bias. Although the training features are relevant to clinical glaucoma assessment, their use in both the predictors and the reference standard limits the independence of the evaluation. Cross-validation does not resolve this limitation. Further evaluation using longitudinal clinical outcomes and independently adjudicated diagnoses is needed to determine whether the model provides diagnostic value beyond the original reference classification. A methodological limitation is that patient-level separation was not enforced during cross-validation. Cross-validation was performed at the record level, and records from the same patient may have appeared in both training and evaluation folds. Although repeated records contained different examination findings, observations from the same patient may remain correlated, which may therefore have resulted in optimistic performance estimates. The reported results could not establish performance in previously unseen patients. More external validation data will be needed to evaluate the performance of the model.

## 5 Conclusions

In summary, we presented a system that combines the DL method and MLCs based on clinical processes. It performed excellently in differentiating early-stage POAG, POAG suspects and healthy eyes. In early POAG patients, POAG suspects and healthy people, VCDR, RNFLt-TI, RNFLt-TS, HCDR, and PSD values play more important roles in the AI model. Although several limitations remain, this model can be helpful in clinical practice. A thorough diagnostic model of early POAG can be established to help ophthalmologists in the primary eye care setting.

## Data Availability

The raw data supporting the conclusions of this article will be made available by the authors, without undue reservation.

## Supplementary material

Supplementary Table 1 Ablation analysis of the SVM using individual modalities and clinical predictors.
