# Supplementary material for: Early diagnosis program for multimodal primary open-angle glaucoma based on clinical process
Source: Front Ophthalmol (Lausanne). 2026 Oct 21;6:1975341. doi: 10.3389/fopht.2026.1975341 (PMC13730968; doi:10.3389/fopht.2026.1975341)
Supplement: Supplementary file 1 [file Table1.docx]

**Early Diagnosis Program for Multimodal Primary Open-Angle Glaucoma Based on Clinical Process**

**Jingyi Ma^1, †^, Bin Lv^2, †^, Yuanyuan Li^1^, Pan Fan^1^, Xu Zhao^2^, Yingtao Zhang^2, *^, Huiping Yuan^1, *^**

^1^The Department of Ophthalmology, the Second Affiliated Hospital of Harbin Medical University, Harbin, Heilongjiang Province, China

^2^School of Computer Science and Technology, Harbin Institute of Technology, Harbin, Heilongjiang Province, China

*** Correspondence:**
Corresponding Author:

Yingtao Zhang, e-mail: yingtao@hit.edu.cn.

Huiping Yuan, e-mail: [yuanhp2013@126.com](mailto:yuanhp2013@126.com).

^†^ These two authors contributed equally to this work.

Table S1. Ablation analysis of the SVM using individual modalities and clinical predictors.

| **Input features** | **Number of features** | **AUC (95% CI)** |
| --- | --- | --- |
| All available features | 13 | 0.9858 (0.9724–0.9961) |
| Fundus-derived CDR only: HCDR and VCDR | 2 | 0.8569 (0.8206–0.8912) |
| OCT only: six-sector RNFL thickness | 6 | 0.9756 (0.9614–0.9877) |
| Visual field only: VFI, MD, and PSD | 3 | 0.7761 (0.7522–0.7970) |
| IOP only | 1 | 0.6218 (0.5742–0.6675) |
| Age only | 1 | 0.6879 (0.6397–0.7334) |
| Age and IOP | 2 | 0.7817 (0.7372–0.8232) |
| All features excluding CDR | 11 | 0.9897 (0.9807–0.9964) |
| All features excluding OCT | 7 | 0.9510 (0.9296–0.9695) |
| All features excluding visual field parameters | 10 | 0.9842 (0.9735–0.9928) |
| All features excluding IOP | 12 | 0.9890 (0.9801–0.9957) |
| All features excluding age | 12 | 0.9820 (0.9708–0.9912) |

**Abbreviations:** SVM, support vector machine; AUC, area under the receiver operating characteristic curve; CI, confidence interval; CDR, cup-to-disc ratio; HCDR, horizontal cup-to-disc ratio; VCDR, vertical cup-to-disc ratio; OCT, optical coherence tomography; RNFL, retinal nerve fiber layer; VFI, visual field index; MD, mean deviation; PSD, pattern standard deviation; IOP, intraocular pressure.
